# To Splice or to Transcribe: SKIP-Mediated Environmental Fitness and Development in Plants

**DOI:** 10.3389/fpls.2019.01222

**Published:** 2019-10-03

**Authors:** Ying Cao, Ligeng Ma

**Affiliations:** College of Life Sciences, RNA Center, Capital Normal University, Beijing, China

**Keywords:** SKIP, alternative splicing, transcriptional regulator, splicing factor, environmental fitness, plant development

## Abstract

Gene expression in eukaryotes is controlled at multiple levels, including transcriptional and post-transcriptional levels. The transcriptional regulation of gene expression is complex and includes the regulation of the initiation and elongation phases of transcription. Meanwhile, the post-transcriptional regulation of gene expression includes precursor messenger RNA (pre-mRNA) splicing, 5′ capping, and 3′ polyadenylation. Among these events, pre-mRNA splicing, conducted by the spliceosome, plays a key role in the regulation of gene expression, and the efficiency and precision of pre-mRNA splicing are critical for gene function. Ski-interacting protein (SKIP) is an evolutionarily conserved protein from yeast to humans. In plants, SKIP is a bifunctional regulator that works as a splicing factor as part of the spliceosome and as a transcriptional regulator *via* interactions with the transcriptional regulatory complex. Here, we review how the functions of SKIP as a splicing factor and a transcriptional regulator affect environmental fitness and development in plants.

## Introduction and Gene Expression Regulation

Due to their sessile nature, plants must respond to both the external environment and internal signals to regulate their environmental fitness and development. To respond to these signals in a precise manner, gene expression must be tightly controlled both temporally and spatially. Gene expression is regulated at multiple levels, but most regulation occurs at the transcriptional and post-transcriptional levels (reviewed in Licatalosi and Darnell, 2010). This allows a gene to be expressed at the correct time, in specific cells, and with the appropriate abundance to support its function.

Transcriptional regulation is crucial for controlling the temporal and spatial expression of a gene, as well as the abundance of precursor messenger RNA (pre-mRNA) molecules. In eukaryotes, messenger RNAs (mRNAs) are transcribed by RNA polymerase II (Pol II) in a complicated process that includes initiation, elongation, and termination steps. The regulation of gene expression at the transcriptional level occurs mainly at the initiation and elongation stages (reviewed in Kwak and Lis, 2013; Jonkers and Lis, 2015; Sainsbury et al., 2015). In the initiation stage, Pol II with an unphosphorylated C-terminal domain (CTD) forms a pre-initiation complex by associating with transcription factors and mediators (reviewed in Hsin and Manley, 2012; Sainsbury et al., 2015; Hantsche and Cramer, 2017). Transcription initiation also requires interactions with *cis*-elements in the genomic DNA sequence and changes in chromatin structure and nucleosome position *via* epigenetic modifications (reviewed in Kouzarides, 2007; Matzke et al., 2009; Bonasio and Shiekhattar, 2014; Voss and Hager, 2014; Sainsbury et al., 2015; Lawrence et al., 2016; Jeronimo and Robert, 2017). The elongation phase is regulated by multiple elongation factors, including Pol II-associated factor 1 complex (Paf1c), and additional factors that influence the epigenetic modification and higher-order structure of chromatin, the phosphorylation status of the Pol II CTD, and the eventual pause and release of Pol II (reviewed in Li et al., 2007; Gilchrist et al., 2010; Hajheidari et al., 2013; Tessarz and Kouzarides, 2014; Van Lijsebettens and Grasser, 2014; Lawrence et al., 2016; Van Oss et al., 2017).

The products transcribed by Pol II from a DNA template require processing to form stable, mature mRNAs. The regulation of pre-mRNA processing at the post-transcriptional level affects the abundance of functional mature mRNAs; thus, it affects both gene expression and function. Post-transcriptional pre-mRNA processing involves 5′ capping mediated by 5′ capping enzymes at the 5′ end of the pre-mRNA, splicing by the spliceosome to remove introns from the pre-mRNA, and 3′ polyadenylation mediated by the 3′ polyadenylation complex at the 3′ end of the pre-mRNA. In addition to these events, pre-mRNA alternative splicing plays a key role in the post-transcriptional regulation of gene expression. By using different splice sites, one pre-mRNA can be processed to multiple transcripts, thus increasing the complexity of the transcriptome and proteome (reviewed in Reddy, 2007; Keren et al., 2010; Syed et al., 2012; Lee and Rio, 2015). Consequently, incorrect splicing of a pre-mRNA can decrease the amount of functional mature mRNA or lead to the production of toxic proteins that may perturb normal cellular processes (reviewed in Reddy, 2007; Braunschweig et al., 2013). The incorrectly spliced variants with a premature termination codon may activate mRNA degradation through the nonsense-mediated decay (NMD) pathway to prevent the formation of nonfunctional or aberrant proteins (reviewed in He and Jacobson, 2015). Therefore, efficient and precise pre-mRNA splicing is crucial to protect gene function (reviewed in Reddy, 2007; Moore and Proudfoot, 2009; Syed et al., 2012; Staiger and Brown, 2013). Accurate splicing of an intron depends on both short consensus sequence elements around the intron and correct assembly of the components of the spliceosome around the intron’s splice sites (reviewed in Wahl et al., 2009; Lee and Rio, 2015).

The spliceosome, which is responsible for pre-mRNA splicing, is a large and highly dynamic protein complex. Specific splicing factors are sequentially recruited to and released from splice sites to mediate efficient splicing. Ski-interacting protein (SKIP), a component of the spliceosome-associated NineTeen complex (NTC), is required to catalyze the first and second transesterification reactions of pre-mRNA splicing in yeast and human cells (Albers et al., 2003; Figueroa and Hayman, 2004; Bessonov et al., 2008; Chen et al., 2011; Schneider et al., 2015; Zhang et al., 2017). In addition, SKIP is a transcriptional coregulator for the expression of some genes in human cells (Laduron et al., 2004; Brès et al., 2005; Brès et al., 2009; Chen et al., 2011). SKIP protein is conserved from yeast to humans including plants. It acts both as a splicing factor to regulate precise and efficient pre-mRNA splicing and as a transcriptional regulator of gene transcription in *Arabidopsis*. This review focuses on the regulatory functions of SKIP that control gene expression at the transcriptional and post-transcriptional levels to mediate the environmental fitness and development of plants.

## Skip Mediates Plant Environmental Fitness by Regulating Alternative Splicing

Flowering is an important developmental phase transition in higher plants. To regulate flowering time, plants integrate endogenous and environmental signals, which are important for survival and crop productivity. To find new components of the flowering time control pathway in *Arabidopsis*, a genetic screen was performed using a T-DNA insertion library for altered flowering time mutants, and a mutant, *eip1-1*, that exhibits an early flowering phenotype under long- and short-day conditions was isolated (Wang et al., 2012). Such a photoperiod-insensitive flowering time defect is characteristic of circadian clock-defective mutants. Consistent with this, *eip1-1* exhibits a lengthened circadian period in a temperature-sensitive manner. Compared to the ∼24-h circadian period of wild-type plants, the circadian period of *eip1-1* is lengthened by ∼2.4 h due to changes in the rhythmic expression of the core oscillator genes *CIRCADIAN CLOCK-ASSOCIATED 1*, *LATE ELONGATED HYPOCOTYL*, and *TIMING OF CAB EXPRESSION 1* (Wang et al., 2012). Map-based cloning revealed that a mutation in At1g77180, which encodes SKIP, is responsible for the flowering time and circadian period defects observed in *eip1-1* (which was therefore renamed *skip-1*). There is a 22-nucleotide deletion at the C-terminus of the *SKIP* locus, which disrupts the integrity of the SKIP protein and impairs SKIP function in *skip-1* plants (Wang et al., 2012).

**Figure 1:**
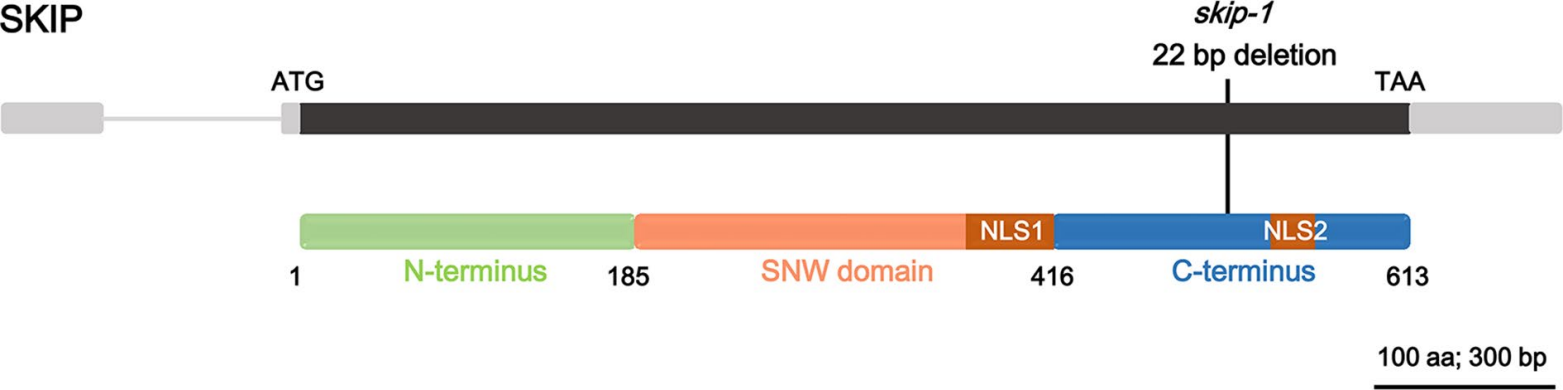
The domain structure of ski-interacting protein (SKIP) and the location of *skip-1* mutant. Gray box: UTR; gray line: intron; black box: exon; colored boxes: domain feature of the protein; black line: the mutation site of the mutant.

SKIP is a single-copy gene in the *Arabidopsis* genome encoding a protein of 613 amino acids with three structural domains: the N-terminus (amino acids 1–185), SNW domain (amino acids 186–416), and C-terminus (amino acids 417–613) (**Figure 1**; Li et al., 2016). The plant SKIP protein sequence is highly similar to that of its ortholog (SKIP) in humans and pre-mRNA processing (Prp)45 in yeast (Wang et al., 2012). SKIP localizes to the nucleus using two nuclear localization signals (NLSs), which lie in the SNW domain and C-terminus, respectively (**Figure 1**; Lim et al., 2010; Li et al., 2016).

In *Arabidopsis*, SKIP co-localizes with the spliceosome components U1 SMALL NUCLEAR RIBONUCLEOPROTEIN-70K (U1-70K) (Golovkin and Reddy, 1996) and SERINE/ARGININE RICH 45 (SR45) (Day et al., 2012) in nuclear bodies (Wang et al., 2012). SKIP associates closely with SR45 and other NTC components, facilitating its integration into the spliceosome (Wang et al., 2012; Li et al., 2016). Mutations in SKIP decrease the splicing efficiency of the spliceosome and can cause genome-wide alternative splicing defects (Wang et al., 2012; Feng et al., 2015). SKIP is required for 5′ and 3′ splice site recognition or cleavage; novel splicing events have been reported in *skip* mutant plants with decreased usage of the dominant GU and AG, respectively, at 5′ and 3′ splice sites (Wang et al., 2012; Feng et al., 2015). Therefore, SKIP is a splicing factor that regulates the efficient and precise splicing of pre-mRNAs on a genome-wide scale in *Arabidopsis*.

In addition, SKIP binds directly to the pre-mRNAs of clock genes, including *PSEUDO-RESPONSE REGULATOR 7* (*PRR7*) and *PRR9*, to regulate their accurate splicing and mRNA maturation (Wang et al., 2012). Compared to wild-type plants, *skip-1* plants show increased levels of aberrantly spliced variants of *PRR7* and *PRR9* and decreased levels of functional, fully spliced *PRR7* and *PRR9* mRNAs. The reduced levels of functional *PRR7 *and *PRR9* mRNAs in *skip-1* contribute to its lengthened circadian period phenotype (Wang et al., 2012). Therefore, SKIP mediates the circadian clock by regulating the alternative splicing of clock genes. These findings demonstrate that post-transcriptional regulation plays vital roles in controlling the circadian clock (Sanchez et al., 2010; Jones et al., 2012; Wang et al., 2012; Li et al., 2019).

SKIP also regulates plant response to abiotic stress (Hou et al., 2009; Zhang Y, et al., 2013; Feng et al., 2015; Li et al., 2016; Li et al., 2019). Mutations in *SKIP* have been shown to cause hypersensitivity to salt or osmotic stress in *Arabidopsis*. Compared to wild-type plants, *skip-1* plants exhibit a significantly decreased germination rate, survival rate, and relative root growth under high-salt or drought conditions (Feng et al., 2015; Li et al., 2016). Meanwhile, ectopic expression of *SKIP* results in increased tolerance to salt or dehydration (Lim et al., 2010). In *Arabidopsis*, salt stress induces genome-wide alternative splicing events, most of which are regulated by SKIP (Feng et al., 2015). SKIP mediates the recognition or cleavage of 5′ alternative donor sites and 3′ alternative acceptor sites, and it is essential for alternative gene splicing under conditions of salt stress (Feng et al., 2015). Transcripts of several salt tolerance-related genes, including *NA^+^/H^+^ EXCHANGER 1* (*NHX1*), *CALCINEURIN B-LIKE PROTEIN 1* (*CBL1*), *DELTA1-PYRROLINE-5-CARBOXYLATE SYNTHASE 1* (*P5CS1*), *RARE-COLD-INDUCIBLE 2A* (*RCI2A*), and *PROTEIN S-ACYL TRANSFERASE 10* (*PAT10*), are aberrantly spliced in *skip-1* under salt stress conditions, decreasing the abundance of fully spliced mRNAs. Premature termination during the translation of these aberrantly spliced variants in *skip-1* reduces the level of functional proteins, resulting in salt hypersensitivity (Feng et al., 2015; Li et al., 2016; Li et al., 2019). Therefore, SKIP is necessary for plants to respond to salt or drought stress, and alternative gene splicing is crucial for plants to respond to environmental cues (Hou et al., 2009; Zhang Y, et al., 2013; Feng et al., 2015; Li et al., 2016; Li et al., 2019; reviewed in Filichkin et al., 2015; Laloum et al., 2018).

In summary, SKIP is a splicing factor that is essential for the precise and efficient splicing of pre-mRNAs on a genome-wide scale, and it mediates the circadian clock and resistance to salt or drought stress by regulating the alternative splicing of clock and salt tolerance-related genes in plants.

## Skip Mediates the Floral Transition by Regulating Transcription

Initially, *skip-1* was characterized as a photoperiod-insensitive early flowering mutant. As defects in the circadian clock may cause changes in the temporal expression of CONSTANS (CO), which regulates *FLOWERING LOCUS T* (*FT*) transcription and affects flowering time (reviewed in Yanovsky and Kay, 2003; Song et al., 2015; Shim et al., 2017), some speculate whether SKIP regulates flowering time by regulating the circadian expression of *CO*. However, no obvious change in the expression of *CO* was observed in *skip-1* (Cao et al., 2015), suggesting that the early flowering phenotype of *skip-1* is not caused by a circadian clock defect.

FLOWERING LOCUS C (FLC) is a MADS-box transcription factor that dose-dependently suppresses the floral transition (reviewed in He and Amasino, 2005; Michaels, 2009; Romera-Branchat et al., 2014; Whittaker and Dean, 2017). Both sense and antisense (*COOLAIR*) transcripts of *FLC* undergo alternative splicing (Marquardt et al., 2014; Mahrez et al., 2016); moreover, temperature-dependent alternative splicing of *FLOWERING LOCUS M* (*FLM*), which changes the ratio of *FLM-β* to *FLM-δ*, plays a vital role in regulating the temperature-dependent floral transition (Lee et al., 2013; Posé et al., 2013). Given that SKIP is a splicing factor that regulates genome-wide pre-mRNA splicing in *Arabidopsis*, it was suggested that SKIP is essential for the alternative splicing of sense and antisense *FLC* transcripts, or *FLM* pre-mRNA, to regulate the level of functional, mature mRNAs in the control of flowering time. However, splicing defects in *FLC* sense and *COOLAIR* transcripts were not observed in *skip-1* (Cao et al., 2015). In addition, even though SKIP is required for the alternative splicing of *FLM*, the alternative splicing pattern of *FLM* pre-mRNA (i.e., the ratio of *FLM-β* to *FLM-δ*) in response to temperature changes was not obviously affected in *skip-1* (Cao et al., 2015). Thus, SKIP is not required for the accurate splicing of *FLC* or *COOLAIR* pre-mRNA. Instead, *FLC* transcription (i.e., the level of unspliced *FLC* mRNA) is significantly repressed in *skip-1*; this is reflected in the obvious repression of mature *FLC* transcripts and the early flowering phenotype observed in *skip-1* under different photoperiod and temperature conditions (Cao et al., 2015; Li et al., 2016; Li et al., 2019). Thus, SKIP activates *FLC* expression at the transcriptional level in a photoperiod- and temperature-independent manner, and it represses flowering time by promoting *FLC* transcription.

To determine how SKIP activates *FLC* transcription, thereby repressing flowering, a yeast two-hybrid screen was performed to identify factors that interact with SKIP. ELF7, a Paf1c component that regulates transcription elongation, was found to interact with SKIP (Cao et al., 2015). Paf1c represses flowering by promoting the trimethylation of histone H3 at lysine 4 on *FLC* chromatin and activating *FLC* transcription (He et al., 2004; Oh et al., 2004; Yu and Michaels, 2010). It was verified that SKIP interacts physically and genetically with ELF7 to regulate flowering time in *Arabidopsis* (Cao et al., 2015; Li et al., 2016). SKIP and ELF7 bind directly to *FLC/MAFs* chromatin and promote histone H2B monoubiquitination, increasing the trimethylation of histone H3 at lysine 4 and *FLC/MAFs* transcriptional activation, thereby repressing the floral transition in wild-type plants (Cao et al., 2015). During this process, SKIP functions as a co-transcriptional activator, mediating plant flowering *via* the regulation of *FLC/MAFs* transcription. Therefore, SKIP can promote the transcription of specific genes as a co-transcriptional activator in plants.

## Conclusion and Prospects

**Figure 2:**
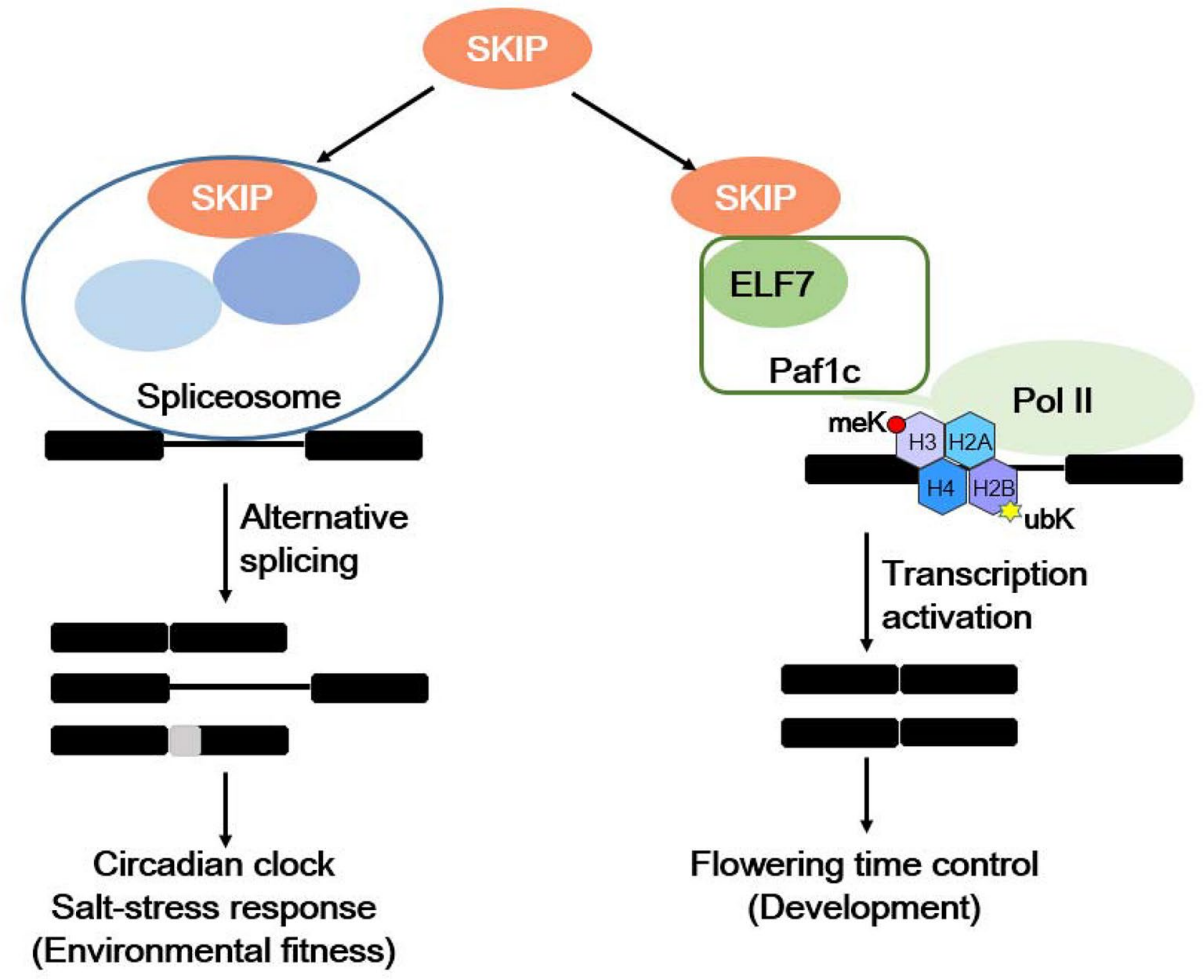
The dual functions of SKIP in mediating environmental fitness and development in plants. SKIP is a splicing factor that regulates the alternative splicing of clock and stress tolerance-related genes in order to mediate the circadian clock and stress responses. It also functions as a transcriptional coactivator by interacting with RNA polymerase II-associated factor 1 complex (Paf1c) to activate *FLC/MAFs* transcription and mediate the floral transition.

Plants respond to internal and environmental signals affecting their development and environmental fitness by accurately regulating gene expression at the transcriptional and post-transcriptional levels. SKIP has a dual function in plants: it acts as a splicing factor to control efficient and precise pre-mRNA splicing on a genome-wide scale by interacting with other spliceosome components (e.g., SR45) and integrating into the spliceosome, and it is required for 5′ and 3′ splice site recognition or cleavage (Wang et al., 2012; Feng et al., 2015). SKIP affects the circadian clock and plant responses to salt stress by regulating the accurate splicing of certain clock- and salt tolerance-related genes (Wang et al., 2012; Feng et al., 2015; Li et al., 2016; Li et al., 2019). SKIP also functions as a transcriptional co-regulator by interacting with other transcriptional regulators (e.g., Paf1c), and it mediates the floral transition (Cao et al., 2015; Li et al., 2016; Li et al., 2019). Therefore, SKIP precisely regulates gene expression at the transcriptional and post-transcriptional levels to mediate plant development and environmental fitness (**Figure 2**).

In addition to SKIP, several other splicing factors are believed to have dual functions in splicing and transcription in *Arabidopsis*. For example, the RNA-binding protein SR45, first identified as an interacting partner of U1-70K and an essential splicing factor in plants (Golovkin and Reddy, 1999; Ali et al., 2007; Carvalho et al., 2016), was reported to be recruited to *FLC* chromatin by VIVIPAROUS 1/ABI3-LIKE factor 1 (VAL1), a transcriptional repressor that further recruits the transcriptional repression complex plant homeodomain–polycomb repressive complex 2 (PHD-PRC2), resulting in decreased histone acetylation of *FLC* chromatin and *FLC* transcriptional silencing during vernalization (Qüesta et al., 2016). This suggests that SR45 is a component of the transcriptional repression complex that suppresses *FLC* expression in *Arabidopsis*. In addition, SR45 participates in a small interfering RNA-directed DNA methylation (RdDM) pathway that mediates gene silencing in *Arabidopsis* (Ausin et al., 2012). ZINC-FINGER AND OCRE DOMAIN-CONTAINING PROTEIN 1 (ZOP1), a pre-mRNA splicing factor, associates with such typical spliceosome components as U1-70K, STABILIZED 1 (STA1, a PRP6-like splicing factor), and RNA-DIRECTED DNA METHYLATION 16 (RDM16, pre-mRNA-splicing factor 3) and is required for RdDM-mediated transcriptional gene silencing (Dou et al., 2013; Huang et al., 2013; Zhang CJ, et al., 2013). Furthermore, PRP31, a conserved pre-mRNA splicing factor, associates with STA1, ZOP1, and RDM16 to regulate transcriptional gene silencing in a manner independent of the RdDM pathway (Du et al., 2015). Therefore, it appears that SKIP is not the only factor required for the regulation of gene expression at both the transcriptional and post-transcriptional levels. Bifunctional splicing factors may provide an effective way for plants to coordinate their responses to environmental and internal signals in order to adjust their development and environmental adaptation *via* accurate gene expression regulation at the transcriptional and post-transcriptional levels.

Resolved structures of the spliceosome from yeast and human cells indicate that SKIP is intrinsically highly disordered; it serves as a scaffold protein and interacts directly with other necessary components to facilitate splicing by promoting spliceosome assembly (Yan et al., 2015; Bai et al., 2017; Zhang et al., 2017; Zhang et al., 2018). It will be interesting to determine the functions of SKIP in transcriptional complex assembly (e.g., Paf1c-SKIP) and transcriptional regulation in plants. In addition, as transcription is usually coupled with splicing (reviewed in Naftelberg et al., 2015; Saldi et al., 2016; Herzel et al., 2017) and given that SKIP is integrated into both the spliceosome and transcriptional complexes (Wang et al., 2012; Cao et al., 2015; Li et al., 2016; Li et al., 2019), it should be investigated whether SKIP mediates the coupling of transcription and splicing in *Arabidopsis*.

## Author Contributions

YC and LM wrote the manuscript.

## Funding

This work was supported by grants from the National Natural Science Foundation of China (31770247 and 31600248) and the Innovation Research Team of Beijing Municipal Government Science Foundation (IDHT20170513).

## Conflict of Interest

The authors declare that the research was conducted in the absence of any commercial or financial relationships that could be construed as a potential conflict of interest.
